# UBE2Q1, as a Down Regulated Gene in Pediatric Acute Lymphoblastic Leukemia

**Published:** 2014

**Authors:** Atefeh Seghatoleslam, Farzaneh Bozorg-Ghalati, Ahmad Monabati, Mohsen Nikseresht, Ali Akbar Owji

**Affiliations:** 1 *Histomorphometry & Stereology Research Center, School of Medicine, Shiraz, Iran.*; 2 *Department of Biochemistry, School of Medicine, Shiraz University of Medical Sciences, Shiraz, Iran.*; 3 *Department of Pathology, School of Medicine, Shiraz University of Medical Sciences, Shiraz, Iran.*; 4 *Cellular and Molecular Research Center, Yasuj University of Medical Sciences, Yasuj, Iran.*

**Keywords:** Ubiquitin-conjugating enzyme, UBE2Q1, QRT-PCR, pediatric acute lymphoblastic leukemia

## Abstract

Ubiquitin - proteasome system (UPS), the major protein degradation pathway in the cells, typically degrades short - lived and damaged proteins and regulates growth and stress responses. This pathway is altered in various cancers, including Acute Lymphoblastic Leukemia (ALL). ALL begins with a change in bone marrow cells and is the most common type of leukemia in children under 15 years. UBE2Q1 as a new characterized gene of E2 enzyme family is located on chromosome 1 and reported to be altered in some malignancies. In this study, we aimed to explore the expression pattern of UBE2Q1 gene in children with ALL. For this purpose, a series of RT - PCR and quantitative RT - PCR were performed on a collection of 20 bone marrow samples of ALL patients and the same number of whole blood samples of age - matched normal subjects. Gel electrophoresis of RT - PCR products revealed the expression of UBE2Q1 mRNA in most of the normal (90%) and about half of the leukemic (45%) samples. QRT - PCR data indicated that only 1 patient out of 20 (5%) showed up regulation of the gene (> 2 folds). In 4 patients (20%), the expression of UBE2Q1 mRNA was equivocal (from 1/2 to 2) and in 15 cases (75%), the gene was down regulated (> 1/2) when compared to the normal samples. In conclusion, down regulation of UBE2Q1 in the majority of the leukemic samples suggests its potential implication in the pathogenesis of ALL. UBE2Q1 can be considered as a molecular marker and a candidate targeting to treat ALL in the future.

Ubiquitin proteasome system (UPS) plays an important role in a variety of cellular functions and homeostasis (1-2). In this system, degradation of targeted proteins starts with covalent attachment of ubiquitin to substrate followed by a series of enzymatic cascade reactions including activating (E1), conjugating (E2) and ligating (E3) enzymes. Substrate - specific E3s, along with the specific E2s, ensure selective protein targeting for proteolysis (3). The 26S proteasome, a large multi-subunit complex of UPS, then degrades the ubiquitin tagged proteins. Different studies have revealed the importance of UPS as a key pathway in the cell cycle such as cell signaling (4), apoptosis (5-6) and growth factor receptor signaling (7). Therefore, alteration in this system could result in many diseases especially cancers. In our previous studies, we characterized the two new members of E2 family, UBE2Q1 and UBE2Q2 genes, and reported their differential expression in some human cancers. Our preliminary study has shown that the newly characterized human gene, UBE2Q1, is differentially expressed in head and neck squamous cell carcinoma (data not published), breast tumor (8) and colon cancer (9). According to NCBI database, this gene is located on chromosome 1q21.3 with an open reading frame (ORF) of 1269 base pairs which encodes a protein composed of 422 amino acids. It contains two structural domains, an ubiquitin conjugate / RWD - like domain at the N - terminal and a ubiquitin conjugating enzyme E2 domain at the C-terminal region. According to NCBI conserved domain database, the C-terminal domain is highly conserved among species (98% similarity of the whole gene to the mouse counterpart), suggesting its important function. A homologue of this gene, UBE2Q2 was also shown to be up regulated in hypo - pharyngeal tumors (10), breast cancer (11), Acute Lymphoblastic Leukemia (12) and colon cancer epithelium (13). The alteration of this gene was also reported in fibroblastoma, glioma and alveolar epithelial carcinoma (14). Pediatric acute lymphoblastic leukemia (ALL), a malignancy with changes in bone marrow cells is the most common type of leukemia in children under 15 years. It originates in a single B or T lymphocyte progenitor (15). In this study we investigated the expression of UBE2Q1 gene in patients with ALL and compared it with the healthy normal subjects.

## Materials and Methods

**Sample preparation**

20 bone marrow aspirates from ALL patients of 3 - 14 years- old were collected for this study. The patients were selected from those who attended the Hematology and Oncology Clinics of Shahid Faghihi Hospital affiliated to Shiraz University of Medical Sciences, Shiraz, Iran. All patients were new cases of ALL and had not taken any therapeutic medication at the time of sampling. In all cases, sample collection was performed according to the rules governed by the Ethics Committee of Shiraz University of Medical Sciences. The collected samples were classified by immune phenotyping performance and were then cell counted. The clinic - pathological significance was determined and the samples were prepared for RNA extraction. Accordingly, 20 peripheral blood samples collected from normal age - matched children were included in the study as healthy controls.

**RNA extraction**

Bone marrow or peripheral blood samples were collected in EDTA. Ficoll density centrifugation was used immediately for the isolation of white blood cells (WBC) from the samples, then the cells were lysed using Tripure isolation reagent (Roche Applied Science, Germany). Total RNA was extracted according to the manufacturer’s instruction. The purity and quantity of total RNA were determined by ultraviolet spectrophotometer. The integrity of RNA was confirmed using agarose gel electrophoresis.

**Reverse Transcriptase Polymerase Chain Reac-tion (RT - PCR)**

Complementary DNA (cDNA) was synthesi-zed from 5 µg of RNase – free DNase treated RNA (random primer or random hexamers) with the Revert Aid first strand cDNA synthesis kit (Fermentas, EU). The primers were designed using primer 3 software in order to amplify exon-exon junction containing regions. The specificity of the primers was verified by Blast analysis at NCBI. The forward and reverse primer sequences were 5'-CTGGCAAGGGTTGATCCAGC-3' and 5'-TCC-TGTCCGCATCCACTGCA-3' for UBE2Q1 gene and 5'-GAAGGCTGTGGTGCTGATGG-3' and 5'-CCGGATATGAGGCAGCAGTT-3' for the ubiqui-tous internal control gene, RPLPO (ribosomal protein, large, PO). PCR conditions were optimized to achieve a sharp and single specific band for all primers as follows: initial denaturation (94°C, 5 min), 30 cycles for amplification (denaturation: 94°C, 30 sec, annealing: 62.5°C, 30 sec, extension: 72°C, 1 min) and final extension (72°C, 7 min). After each run, agarose gel electrophoresis (1.5% gel, 100 volts) in the presence of gel red (Biotiom, USA) along with a DNA marker of 50-1000 bp (Fermentas, EU) was performed for visualizing the PCR products. For this purpose, 12 µl of each PCR product was mixed with 5µl of loading dye and loaded on the gel. A single specific band was detected for each of the UBE2Q1 and RPLPO genes, as expected.

**Quantitative Real time PCR**

For quantitative reverse transcriptase polymerase chain reaction experiments ABI thermal cycler (Applied Biosystems, USA) was applied. The PCR reaction mixture included 2 µl of cDNA (~150 ng), 1 µl of 10 µM solutions of each of the forward and reverse primers, along with 12.5 µl of ABI SYBR Green super Mix (ABI, USA) in a total volume of 25 µl. The samples were loaded in duplicates or triplicates. The procedure was followed by amplifications including an initial denaturation at 94°C for 5 min, 45 cycles (30 sec at 94°C, 30 sec at 58°C and 1 min at 72°C) and final extension at 72°C for 7 min followed by a melting curve (58-95°C) analysis. To generate a standard curve, a set of five - fold serial dilutions of the internal standard was applied for all runs. Agarose gel (20% w/v) electrophoresis of the amplified products together with melting curve analysis (Tm) was used to confirm the product specificity. All quantitative real-time RT - PCR assays were linear within this concentration range, with correlation coefficients of r^2^> 0.999. Data were analyzed by the ratio=2^-∆∆ct^ method (16). Relative amounts of UBE2Q1 mRNA were normalized to the levels of RPLPO mRNA for each sample. Statistical analysis of the differences in the UBE2Q1 mRNA expression levels was done by Mann-Whitney rank-sum test using SPSS10 analytic software (SPSS, Inc, Chicago). The differences were considered significant at p< 0.05.

## Results

**Clinicopathological data**

Table 1 represents a brief clinical history, examination findings and molecular subgroups of 20 patients with ALL. The ratio of UBE2Q1 mRNA expression level in the bone marrow of the patients compared to normal controls is indicated in the right column. As seen, these quantities varied between about zero and 3.26. The major laboratory findings of peripheral blood samples related to 20 normal individuals as mean ± standard error of the mean (Mean±SEM) are presented as follows: White Blood Cells×10^3^/ µl = 11.43 ± 0.66; Red Blood Cells×10^6^/ µl = 4.22±0.13; Platelets count×10^3^/ µl = 321 ± 26.34; and Hemoglobin = 12.24 ± 0.30 (g/dl).

**UBE2Q1 expression in ALL patients and normal peripheral blood samples by RT-PCR**

**Fig. 1:**
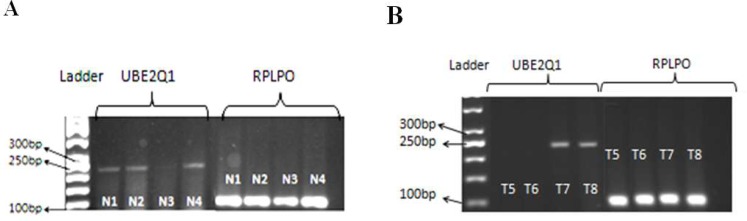
Electrophoresis of reverse transcriptase - polymerase chain reaction products of the UBE2Q1 gene in A. normal subjects (left panel) and B. ALL patients (right panel). Internal control, RPLPO bands are presented in lanes 5- 8 in both left and right panels. Lanes 1 and 2 in the right panel and lane 3 in the left panel represent two ALL and one normal samples without any detectable UBE2Q1 mRNA expression and very low intense band, respectively

Reverse transcriptase polymerase chain reaction using specific primers for UBE2Q1 gene was performed on samples from bone marrow cells of ALL patients and peripheral blood cells of normal subjects. As shown in Figure 1 (A and B) a single specific band of 229 bp for UBE2Q1 and 102 bp for RPLPO gene (as internal control) was observed. Electrophoresis of the gene expression product showed that 9 out of 20 leukemic bone marrow samples (45%) and 18 out of 20 normal peripheral blood samples (90%) expressed UBE2Q1 mRNA at a detectable level.

**Evaluation of UBE2Q1 gene expression in pediatric acute lymphoblastic leukemia by Quantitative RT-PCR**

**Fig. 2:**
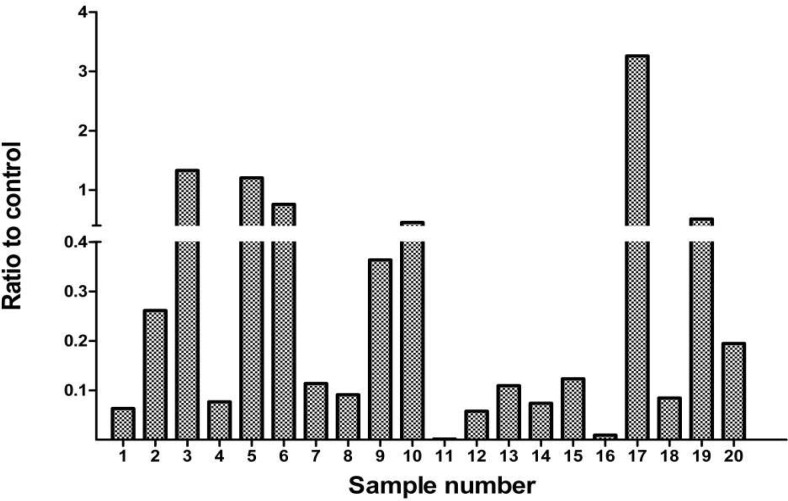
The ratios of the expression of UBE2Q1 mRNA of ALL samples to that of normal controls obtained by quantitative RT-PCR using specific pairs of primers. RPLPO was used as internal control

To quantify the amount of UBE2Q1 gene expression in both leukemic and normal samples, a series of QRT-PCR were performed on the collection of 20 bone marrow samples of ALL patients and 20 whole blood samples of normal children. In the 20 cases investigated, only 1 out of 20 (5%) showed up regulation (>2 fold) of the gene. In 4 samples (20%), the expression of UBE2Q1 was equivocal (between 1/2 and 2) and in 15 out of 20 (75%), the down regulation of the gene (>1/2) was observed. The ratios of UBE2Q1 gene expression of ALL samples to that of normal controls are presented in Figure 2.

**Table 1:** Hematological features and molecular subgroups of patients with acute lymphoblastic leukemia and the ratio of UBE2Q1 mRNA levels in their bone marrow cells compared to normal controls

**NO**	**Age**	**WBC** a **× 10** ^3^ **/μl**	**RBC ** b **×10** ^6^ **/μl**	**HB** c **(g/dl)**	**PLT** d **× 10** ^3^ **/μl**	**Blast(%)**	**Lym** e **(%)**	**Immunophenotype (BM)**	**Ratio**
T_1_	13	102.9	2.13	06.4	55	97	2	ALL (early pre B cell)	0.063
T_2_	10	385.0	3.35	07.8	17	93	3	ALL (pre B cell)	0.260
T_3_	3	184.2	3.37	09.1	85	85	9	ALL (T cell )	1.320
T_4_	5	240.0	3.10	07.1	45	90	4	ALL (Common type)	0.070
T_5_	5	014.4	3.11	10.9	76	74	26	ALL (T cell )	1.200
T_6_	8	086.2	2.99	08.0	50	97	3	ALL (T cell )	0.750
T_7_	11	250.0	3.21	07.5	21	95	4	ALL (pre B cell)	0.110
T_8_	6	200.0	2.24	06.8	59	89	8	ALL (Common type)	0.090
T_9_	5	150.5	3.20	08.9	45	96	3	ALL (pre B cell)	0.360
T_10_	6	100.0	2.10	06.1	28	95	4	ALL (Common type)	0.450
T_11_	14	160.0	3.44	04.5	120	90	5	ALL (pre B cell)	0.000
T_12_	5	164.1	2.11	06.1	76	96	3	ALL (T cell )	0.050
T_13_	8	500.0	3.20	07.0	46	89	8	ALL (pre B cell)	0.100
T_14_	7	287.0	2.12	05.9	84	93	5	ALL (Common type)	0.070
T_15_	5	095.5	2.15	06.2	36	96	4	ALL (T cell )	0.120
T_16_	6	380.0	2.56	08.1	49	92	5	ALL (Common type)	0.000
T_17_	8	250.0	3.24	08.7	48	91	6	ALL (Common type)	3.260
T_18_	5	286.0	3.20	08.5	79	97	2	ALL (pre B cell)	0.080
T_19_	9	070.0	2.15	07.0	46	87	9	ALL (pre B cell)	0.510
T20	10	026.6	2.18	11.4	35	88	8	ALL (L3)	0.190

a : White Blood Cell,

b :Red Blood Cell,

c :Hemoglobin,

d :Platelet count,

e :Lymphocyte,

T :Test(ALL sample)

## Discussion

The goal of this study was to investigate the expression pattern of the newly characterized human gene, UBE2Q1 in ALL patients. UBE2Q1 protein contains a predicted highly conserved domain at its carboxy terminal region, UB-CON, suggesting its important catalytic function (17). The amino terminal domain of this gene contains an RWD - domain, which is included in about 100 proteins (17). This domain is suggested to be involved in protein– protein interactions and substrate recognition (18). In our previous studies we showed that UBE2Q1 is up regulated at mRNA level in head and neck squamous cell carcinoma (unpublished data), and at both protein and mRNA levels in breast (8) and colon cancers (9). UBE2Q2, a homologue of this gene, is also up regulated at protein and/ or mRNA levels in different cancers (10-13). We also showed the alteration of cell cycle progression in cultured cells by UBE2Q2 (19). Differential expression of other components of ubiquitin-proteasome pathway in leukemia has been assessed in several studies. Down regulation of ubiquitin gene expression during the differentiation of human leukemia cells (20), over expression of CDC34 (21), UbcH5A and UbcH6 (22), E2N (CCRF-CEM) in acute lymphoblastic leukemia (23), induction of UBCH8 in leukemic patients treated with chemotherapy (24) and differential expression of UBE2N and UBE2C (also called UbcH10) between different types of lymphoma cell lines and hematological malignancies (25-27) are among the numerous examples of the involvement of ubiquitin conjugating enzymes in leukemia development. Important cell pathways such as DNA repair, gene regulation, protein degradation and translocation, apoptosis and immune response are reported to be affected by ubiquitination and de - ubiquitinition processes (28-29). Gene expression analysis can identify potential therapeutic targets for ALL as well as identifying patients with favorable and unfavorable early responses to therapy (30). The results of these studies predicted treatment failure in T-ALL patients (31) in addition to the early response and outcome in high risk childhood ALL (32). The clinical variables such as age and number of WBC at diagnosis, the rate of response to induction and other factors have also been studied in parallel (33). All these together could help to apply earlier intensified or alternative treatments. Considering the important role of proteasome and its upstream system of ubiquitin conjugating enzymes in cancer, it is important to identify the members of this family of enzymes that show differential expression in malignancies. Such enzymes can be used as potential molecular markers for malignancies including leukemia.

In this study, we primarily evaluated the expression pattern of the UBE2Q1 mRNA in normal and leukemic cells (ALL). Our RT- PCR results revealed the expression of UBE2Q1 mRNA in 45% of leukemic cells and 90% of normal bone marrow samples. In other words, while most of the normal samples revealed the expression UBE2Q1 gene, the expression was detectable in only 45% of the leukemic samples. To confirm these data, we employed Real- time RT - PCR to quantify the expression of UBE2Q1 gene in the same ALL and normal samples. Thus, down regulation of UBE2Q1 gene was observed in 75% of ALL samples by more than one- half when compared to the mean expression levels of UBE2Q1 mRNA in the normal peripheral blood cells tested positive for the transcript. Although this finding is dissimilar to those of our previous studies indicating the up regulation of UBE2Q1 in breast and colorectal carcinomas, however, this may suggest a different way of involvement of this gene in ALL development.

In the present study, there are some points that can affect the results. First of all the source of the cells collected from peripheral blood samples of healthy controls mainly contained mature and differentiated cells, whereas, the mononuclear cells isolated from bone marrow of ALL patients were predominantly immature leukemic blast B or T cells. The second issue to be considered was the obligation of collecting samples of cancerous and normal cells from separate individuals. Although the more appropriate matched control is normal bone marrow, we had no luck to find normal volunteers and ethically we were not able to collect such samples, particularly from children. Even bone marrow aspirates from patients with other diseases was not an ideal normal control for this study as there is no information about the potential influence of other diseases on the expression of UBE2Q1 gene. However, because of the novelty of the gene and to have a better understanding about it, we found it worthy to be studied. 

UBE2Q1 is a newly characterized gene, therefore, the hypothetic role identified for this gene product as ubiquitin conjugating enzyme needs to be confirmed. Identification of the potential substrate(s) for its protein, finding its link with cancer development of different origin and its ultimate role in cancer is as potential outlines for further studies. However, while the necessity of extending investigation on this gene and on a larger number of samples still exists, our data suggest that the novel human gene, UBE2Q1, might be potentially a useful molecular marker and a candidate for drug targeting to treat ALL in the future.
